# The effects of emotional distress on the postoperative survival and progression in patients with breast cancer: a prospective observational study

**DOI:** 10.1186/s12957-025-04162-w

**Published:** 2025-12-30

**Authors:** Shanqing Xu, Li Yang, Juan Zhang, Aihua Tan, Dabao Xu, Jianbin Tong

**Affiliations:** 1 Hunan Province Key Laboratory of Brain Homeostasis, Third Xiangya Hospital, Central South University, Changsha, Hunan 410013 P.R. China; 2 Department of Anesthesiology, The Third Xiangya Hospital, Central South University, Changsha, Hunan 410013 P.R. China; 3 Third Xiangya Hospital, Central South University, Changsha, Hunan 410013 P.R. China; 4 Department of Breast Surgery, Tangshan People’s Hospital, Tangshan, Hebei China; 5 Department of Anesthesiology, Third Xiangya Hospital, 138 Tongzipo Road, Yuelu District, Changsha, Hunan 410013 P.R. China; 6 Department of Gynecology, Third Xiangya Hospital, 138 Tongzipo Road, Yuelu District, Changsha, Hunan 410013 P.R. China

**Keywords:** Breast cancer, Emotional distress, Depression, Prognosis, Anxiety

## Abstract

**Background:**

Emotional distress (ED), characterized by symptoms of depression and anxiety, is highly prevalent in patients with breast cancer. Although retrospective studies suggest an association between ED and adverse cancer outcomes, findings from prospective studies remain inconsistent.

**Objective:**

This prospective observational study aimed to investigate the association between emotional distress and long-term survival outcomes in patients with breast cancer following curative surgery.

**Methods:**

A total of 159 patients with breast cancer were enrolled. Baseline levels of emotional distress were assessed using standardized scales. Patients were prospectively followed for up to 7 years to monitor overall survival (OS) and progression-free survival (PFS). Inverse probability of treatment weighting (IPTW) was employed to adjust the baseline confounders.

**Results:**

The unadjusted 7-year OS and PFS rates in patients with ED were numerically lower than those without ED (OS: 78.5% vs. 93.7%; PFS: 76.9% vs. 89.3%). After adjustment using IPTW, emotional distress was independently associated with lower OS (adjusted HR = 6.52, 95% CI: 1.78–23.89, *P* = 0.005) and PFS (adjusted HR = 3.73, 95% CI: 1.19–11.71, *P* = 0.024).

**Conclusion:**

This study provides prospective evidence that emotional distress is associated with reduced survival and an increased risk of disease progression in breast cancer patients following curative surgery. These findings highlight the importance of integrating psychological assessment and supportive care into routine oncologic management.

**Supplementary Information:**

The online version contains supplementary material available at 10.1186/s12957-025-04162-w.

## Introduction

 Breast cancer is the most commonly diagnosed cancer in women [1, 2]. According to the WHO statistics in 2022, the incidence of breast cancer varies between 26.7 in 100 000 and 100.3 in 100 000 [2]. Despite significant improvements in clinical outcomes owing to advances in early detection and treatment [1, 3, 4], breast cancer still accounts for approximately 7% of all female cancer deaths [5]. Consequently, further improving the long-term prognosis of patients has become a major focus of contemporary breast cancer research.

To date, a number of factors have been reported to affect the recurrence and metastasis of breast cancer after curative surgery [1, 3, 6–9]. Among them, histological subtypes, the status of lymph nodes, and tumor size are well-known risk factors [10–12]. Emotional distress, commonly characterized by symptoms of depression and/or anxiety, is common in patients with breast cancer [13–15]. Its prevalence among breast cancer patients is estimated to be 15 to 54% [16, 17]. Although most women with breast cancer believe that emotional distress has an important influence on the recurrence of their cancer, inconsistent results between retrospective studies and prospective studies have been reported [13, 18]. Population-based retrospective studies have showed that emotional distress is associated with an increased risk of mortality and recurrence of breast cancer. In contrast, a population-based prospective cohort study showed no significant effect of emotional distress on the distant disease-free survival and overall survival of breast cancer patients during median follow-up of 8.2 years (0.8 to 14.4 years) [8]. Another prospective case-control study of 34 breast cancer patients during follow-up of 25 years supported a predictive role of emotional distress for relapse-free survival [19]. These inconsistent findings suggest that (1) the relationship between the prognosis of breast cancer and emotional distress after surgery remains unclear, (2) a relatively short follow-up period in some studies may not be sufficient to observe the long-term impact on survival outcomes, given the high 5-year survival rates of breast cancer (77.5%-90.3%).

To test the relationship between the prognosis of breast cancer and emotional distress after surgery, we evaluated the levels of postoperative emotional distress, and prospectively detected the survival and recurrence of 159 patients with breast cancer(recruited from 1st May 2018 to 8th October 2019)during follow-up of 7 years.

## Materials and methods

### Study design and population

This prospective observational cohort study was conducted as a post-hoc follow-up of a previously completed randomized, double-blind, placebo-controlled trial, which evaluated the effect of probiotic supplementation on cognitive function in breast cancer patients undergoing neoadjuvant chemotherapy [20]. All participants had completed neoadjuvant chemotherapy prior to enrollment in the present study. The original RCT enrolled 160 eligible patients (stage I-III breast cancer, aged 20–60 years, no immune diseases), who were randomly assigned to receive either probiotics or placebo. The present study was a post-trial observational follow-up study focusing on the association between baseline emotional distress and long-term survival, irrespective of the original intervention. One patient was lost to follow-up, resulting in 159 patients included in the final analysis. All patients were followed until September 1, 2025, with a median follow-up of 7 years (range: 1.5–7 years). The study protocol was approved by the Ethics committee of Third Xiangya Hospital, Central South University, Changsha, China(2020-S289).

### Definition of emotional distress

The Self-rating Anxiety Scale (SAS), a validated 20-item instrument with established reliability and validity in Chinese cancer populations, was used to assess the severity of anxiety symptoms. Each item is rated on a 4-point Likert scale, with responses ranging from 1 (“none of the time”) to 4 (“most of the time”). Total scores are computed by summing individual item scores, resulting in a range of 20 to 80 [21, 22]. Consistent with validated diagnostic thresholds for clinical research [21, 22], a total SAS score ≥ 50 was defined as clinically significant anxiety. Similarly, the Self-rating Depression Scale (SDS)—a 20-item tool with comparable reliability and validity in the target population—was used to evaluate depressive symptom severity, employing the same 4-point Likert scoring system as the SAS (total score range: 20–80) [23, 24]. A total SDS score ≥ 53 was defined as clinically significant depression, in accordance with validated cutoff criteria [23, 24]. Emotional distress (ED) was defined as the presence of either clinically significant anxiety (SAS ≥ 50) or clinically significant depression (SDS ≥ 53), aligning with widely accepted standards for clinical distress in oncology settings [21, 23].

### Data collection and outcomes

Follow-up data were primarily extracted from outpatient and inpatient hospital charts. For patients without recent hospital visits, information was collected via structured telephone interviews or WeChat. All endpoint events—death and disease progression—were adjudicated by an independent Clinical Events Committee, which remained blinded to the original treatment allocation. The primary outcome was progression-free survival (PFS), defined as the time from baseline to the first occurrence of disease progression or death from any cause. The secondary outcome was overall survival (OS), defined as the time from baseline to death from any cause.

### Statistical analysis

Given that the classification of emotional distress (ED) was non-randomized and baseline differences were observed between the ED (*n* = 27) and non-ED (*n* = 132) groups, all analyses were conducted as observational. Categorical variables are reported as frequencies and percentages, while continuous variables are presented as medians with interquartile ranges (IQRs) due to non-normal distribution (assessed via Shapiro-Wilk test, data not shown). Baseline between-group comparisons were performed using the Mann–Whitney U test for continuous variables and Fisher’s exact test for categorical variables (due to small expected cell counts in some categories).

To ensure the rigor and reproducibility of results, statistical analyses were performed in a hypothesis-driven sequential framework, with each step focused on validating key assumptions or mitigating potential biases: (1) Validation of core survival model assumptions: For the log-rank test, Schoenfeld residuals derived from the univariate Cox proportional hazards (PH) model (its statistical equivalent) were used to verify the PH assumption (*p* > 0.05). For multivariate Cox models, variable-specific and global Schoenfeld tests confirmed that all covariates satisfied the PH assumption (*p* > 0.05)—a critical prerequisite for reliable hazard ratio (HR) estimates (Supplementary Tables S1 and S2). (2) Evaluation of inverse probability of treatment weighting (IPTW): IPTW was assessed across four key dimensions to minimize bias and ensure robustness: weight stability (summarized via descriptive statistics and histograms), covariate balance (standardized mean difference [SMD] < 0.3 and *p* > 0.05), appropriate propensity score specification (≥ 10 events per covariate), and absence of multicollinearity (variance inflation factor [VIF] < 5) (Supplementary Tables S3–S7). (3) Testing for multicollinearity: Multicollinearity in multivariate Cox models was evaluated using the VIF function from R’s car package. A VIF < 5 confirmed no substantial multicollinearity, ensuring accurate quantification of each variable’s independent association with survival outcomes (Supplementary Table S8). (4) Sensitivity analysis for time-dependent effects: Time-dependent Cox models (incorporating interaction terms of covariates [TNM stage and molecular subtype] with log(time + 20)) were used to address potential PH violations. These models were IPTW-weighted and adjusted for confounders to verify the robustness of primary findings (Supplementary Table S9). (5) Estimation of survival rates: Kaplan–Meier methods were used to estimate 1-, 3-, 5-, and 7-year PFS and OS rates. Logit transformation was applied to calculate 95% confidence intervals (CIs), which quantified uncertainty and enhanced the interpretability of long-term outcomes (Supplementary Table S10).

## Results

### Study baseline assessment

A total of 159 patients were included in the final analytical cohort. Using the predefined criteria (SAS ≥ 50 or SDS ≥ 53), 27 patients (17.0%) were classified as having baseline emotional distress (ED), and 132 patients (83.0%) were categorized into the non-ED group. The baseline clinical and demographic characteristics of the cohort, stratified by ED status, are summarized in Tables 1 and 2. The median age was 46.0 years (IQR: 39.0–52.0), and the median body mass index (BMI) was 23.05 kg/m² (IQR: 20.89–24.83). Comparative analysis revealed no significant differences in most baseline characteristics between the ED and non-ED groups, except for anxiety and depression scores, which were significantly higher in the ED group (both *P* < 0.001), thereby validating the classification scheme (Table 1).

**Table 1:** Baseline demographic and clinical characteristics of the follow-up cohort

Variable	Overall (*n* = 159)	No ED (*n* = 132)	ED (*n* = 27)	*P*
Age, median (IQR), year	46.00 (39.00,52.00)	46.00 (39.75,53.00)	45.00 (38.00,49.00)	0.228
BMI, median (IQR), kg/m²	23.05 (20.89,24.83)	23.04 (20.79,24.75)	23.23 (21.52,24.87)	0.943
TNM Stage, n (%)				0.014
І	48 (30.20)	34 (25.80)	14 (51.90)	
П/Ш	111 (69.80)	98 (74.20)	13 (48.10)	
Molecular subtype, n (%)				0.728
HER-2- Negative	87 (54.70)	70 (53.00)	17 (63.00)	
HER-2-Postive	51(32.10)	44(33.33)	7(25.92)	
Tri-Negative	21(13.20)	18(13.63)	3(11.11)	
Treatment, n (%)				0.699
Placebo	79(49.7)	67(50.76)	12(44.44)	
Probiotics	80(50.3)	65(49.24)	15(55.56)	
Anxiety score, median (IQR)	35.00 (30.00,40.00)	34.00 (30.00,38.00)	48.00 (43.50,53.00)	< 0.001
Depression score, median (IQR)	39.00 (34.00,45.50)	36.00 (34.00,43.00)	54.00 (50.50,59.00)	< 0.001

**Table 2:** Associations between emotional distress and survival analysis

	1-years, n (%), 95%CI	3-years, n (%), 95%CI	5-years, n (%), 95%CI	7-years, n (%), 95%CI
PFS survival rate				
Depression				
No	140[100% (100.0-100.0)]	132[94.6% (91.0-98.3)]	130[93.3% (89.3–97.4)]	125[89.7% (84.9–94.8)]
Yes	20[100% (100.0-100.0)]	16[78.3% (55.5–100.0)]	14[66.7% (43.7–100.0)]	14[66.7% (43.7–100.0)]
Anxiety				
No	146[100% (100.0-100.0)]	137[94.1% (90.5–97.9)]	135[92.2% (88-96.5)]	130[88.8% (83.9–94.0)]
Yes	12[100% (100.0-100.0)]	11[88.1% (68.8–100.0)]	11[85.5% (65.8–100.0)]	11[85.5% (65.8–100.0)]
ED				
No	132[100% (100.0-100.0)]	124[94.5% (90.8–98.3)]	123[93.1% (89-97.3)]	118[89.3% (84.3–94.6)]
Yes	27[100% (100.0-100.0)]	23[84.6% (67.3–100.0)]	21[76.9% (58.5–100.0)]	21[76.9% (58.5–100.0)]
OS survival rate n				
Depression				
No	140[100% (100.0-100.0)]	134[96% (92.8–99.2)]	134[96% (92.8–99.2)]	131[93.9% (90.1–97.9)]
Yes	20[100% (100.0-100.0)]	16[78.3% (55.5–100.0)]	16[78.3% (55.5–100.0)]	14[68.8% (45.5–100.0)]
Anxiety				
No	146[100% (100.0-100.0)]	139[95.4% (92.2–98.8)]	139[95.4% (92.2–98.8)]	135[92.8% (88.7–97.0)]
Yes	12[100% (100.0-100.0)]	11[88.1% (68.8–100)]	11[88.1% (68.8–100.0)]	11[88.1% (68.8–100.0)]
ED				
No	132[100%(100.0-100.0)]	126[95.9% (92.7–99.2)]	126[95.9% (92.7–99.2)]	123[93.7% (89.8–97.8)]
Yes	27[100% (100.0-100.0)]	23[84.6% (67.3–100.0)]	23[84.6% (67.3–100.0)]	22[78.5% (60.0-100.0)]
*95%CI* 95% confidence interval, *PFS* progression-free survival, *OS* overall survival, *ED* Emotional Distress				

Cumulative survival rates were estimated using the Kaplan-Meier product-limit method based on weighted data. Here, "n" denotes the weighted expected number of survivors (not the original actual patient count)

## Survival analysis

**Fig. 1:**
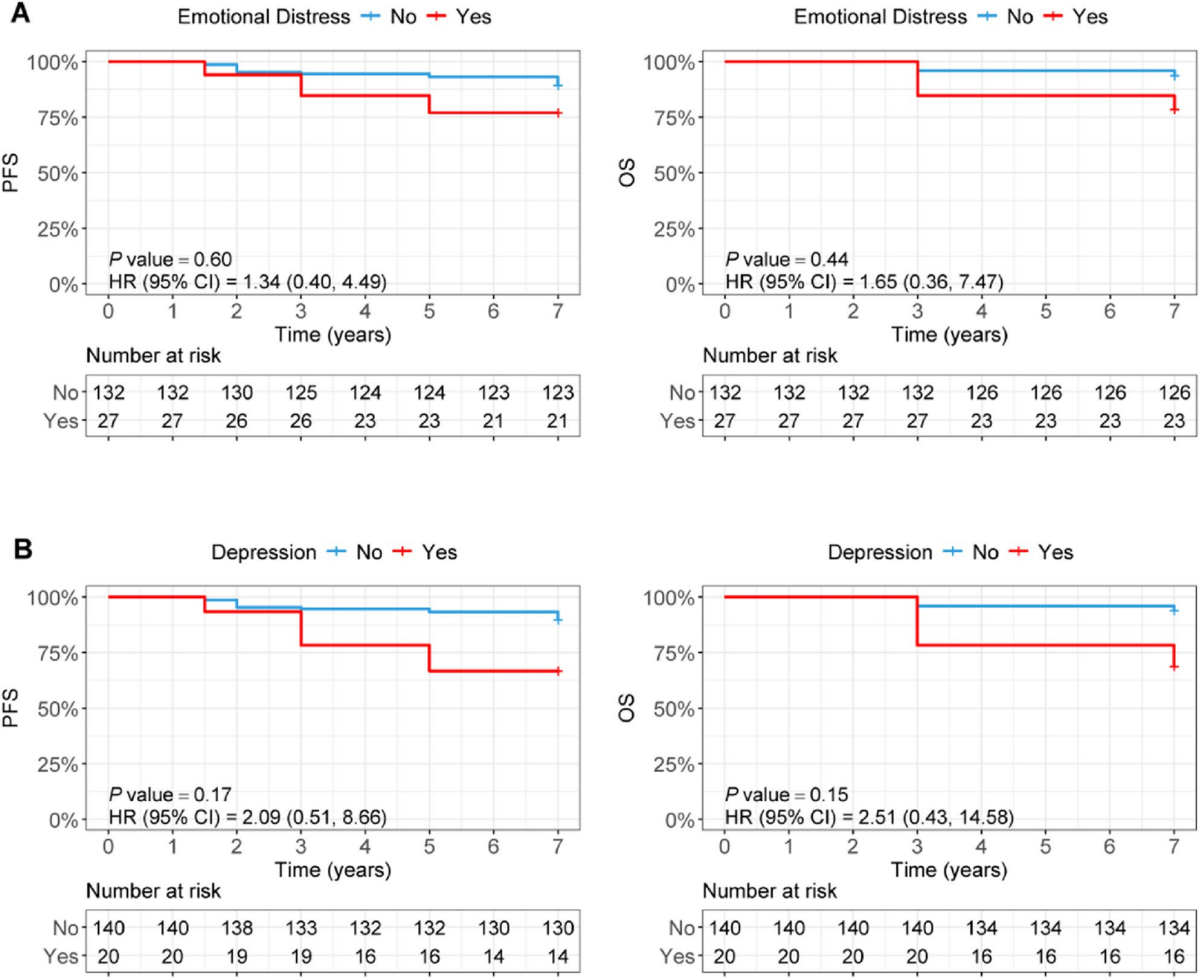
Kaplan-Meier curves for progression-free survival (PFS) and overall survival (OS) stratified by baseline Emotional distress (ED) status **A** and Depression **B**

The endpoints were progression-free survival (PFS) and overall survival (OS). Kaplan-Meier methods with log-rank tests were used to compare 1-, 3-, 5-, and 7-year PFS and OS rates between the ED and non-ED groups, showing that patients with ED had consistently lower survival rates across all time points (7-year PFS: 76.9% vs. 89.3%; 7-year OS: 78.5% vs. 93.7% in the non-ED group) and Kaplan-Meier curves illustrated an inferior trend in PFS and OS for the ED group, though the unadjusted log-rank test was not statistically significant (*P* > 0.05; Table 2 and Fig. 1). To address baseline imbalances (e.g., TNM stage), inverse probability of treatment weighting (IPTW) was applied to adjust for covariates including intervention group (probiotics vs. placebo), age, BMI, molecular subtype, and TNM stage, and after adjustment, ED was independently associated with a significantly increased risk of OS events (adjusted HR = 6.52, 95% CI: 1.78–23.88, *P* = 0.005) and PFS events (adjusted HR = 3.73, 95% CI: 1.18–11.71, *P* = 0.024; Table 3). All statistical models met underlying assumptions: the proportional hazards (PH) assumption was verified via Schoenfeld residuals (*P* > 0.05 for all covariates; Supplementary Tables S1 and S2), and IPTW adjustment achieved adequate covariate balance (standardized mean difference < 0.3) with stable weights and no multicollinearity (variance inflation factor < 5; Supplementary Tables S3–S7), confirming result robustness. Sensitivity analyses using time-dependent Cox models (incorporating interaction terms of TNM stage/molecular subtype with log(time + 20)) to address potential PH violations showed that after adjusting for time-varying effects, confounders, and IPTW weighting, depression remained independently associated with worse PFS (HR = 5.25, 95% CI: 1.54–17.87, *P* = 0.008) and OS (HR = 8.42, 95% CI: 2.45–28.94, *P* = 0.001), ED was significantly associated with reduced PFS (HR = 3.69, 95% CI: 1.04–13.15, *P* = 0.044) and OS (HR = 6.47, 95% CI: 1.78–23.51, *P* = 0.005), while anxiety (SAS ≥ 50) showed no significant association with PFS (HR = 2.37, 95% CI: 0.46–12.34, *P* = 0.305) or OS (HR = 2.51, 95% CI: 0.78–8.07, *P* = 0.124), with detailed results presented in Supplementary Tables S9 and S10.

**Table 3:** Associations between emotional distress and survival outcomes (based on IPTW model)

	PFS			OS		
Group	Unadjusted HR (CI 95%)	Adjusted HR (CI 95%)	Adjusted *P*	Unadjusted HR (CI 95%)	Adjusted* HR (CI 95%)	Adjusted^*^ *P*
Depression						
No	3.75 (1.20,11.67)	5.30 (1.84,15.31)	0.002	5.86 (1.65,20.82)	8.48 (2.50,28.63)	0.001
Yes						
Anxiety						
No	1.39 (0.23,8.50)	2.38 (0.42,13.60)	0.329	1.72 (0.22,13.62)	2.51 (0.75,8.35)	0.134
Yes						
ED						
No	2.36 (0.75,7.45)	3.73 (1.18,11.71)	0.024	3.72 (1.04,13.34)	6.52 (1.78,23.88)	0.005
Yes						

*IPTW* Inverse probability of treatment weighting

## Discussion

The belief that cancer outcomes might be related to psychological factors dates back to around 200 AD when Galen hypothesized that melancholic women were predisposed to breast cancer [8]. Compared to patients with other cancers, patients with breast cancer are more likely to experience emotional distress because of concerns about altered body image, subsequent treatment, cancer recurrence, as well as economic burden. However, to date, the relationship between the prognosis of breast cancer and emotional distress after surgery remains unclear.

In this prospective observational study, we found that breast cancer patients with baseline emotional distress had significantly worse long-term survival outcomes after adjusting for key clinical confounders using IPTW. Our findings align with several previous studies reporting that emotional distress is associated with higher cancer-specific mortality and poorer overall survival [13, 25–27]. Our findings are different from a population-based prospective cohort study that showed no significant effect of emotional distress on the distant disease-free survival and overall survival of breast cancer patients during median follow-up of 8.2 years (0.8 to 14.4 years). A possible explanation for the difference is that the patients in our study were all recruited over a short period in a single medical center, ensuring more homogeneous treatments than the population-based prospective cohort study. Collectively, our study supports that emotional distress promotes the progression and metastasis of breast cancer after surgery.

The mechanisms by which emotional distress promotes the progression and metastasis of breast cancer after surgery is still unclear. Previous studies have indicated that emotional distress plays a potential role in fostering the development of an immunosuppressive tumor microenvironment via inducing the dysregulation of the hypothalamic-pituitary-adrenal (HPA) axis. New evidence have showed that β-adrenergic signaling can suppress an effector phenotype in CD8 + T Cells and undermines checkpoint Inhibitor therapy [28]. Stress-glucocorticoid-TSC22D3 axis compromises therapy-induced antitumor immunity [29]. kisspeptin/GPR54 signaling plays an important role in the stress-induced tumor immune evasion [30]. These new findings support that emotional distress promotes the recurrence and metastasis of breast cancer after surgery possibly via complex molecular signaling of the HPA axis downstream.

Several limitations of our study should be acknowledged. First, the relatively small sample size, particularly the limited number of patients with ED, may limit the statistical power and increase the risk of type II errors, despite the use of robust statistical adjustment. Second, although we adjusted for several key confounders, residual confounding from unmeasured factors cannot be entirely ruled out. Third, ED was measured only at baseline (no longitudinal assessments during follow-up), precluding capture of dynamic ED changes—may have oversimplified the ED-survival relationship, future studies should use serial ED measurements to address this gap. Finally, the single-center design may affect the generalizability of our findings to broader breast cancer populations.

In conclusion, this prospective study provides evidence that emotional distress is independently associated with an increased risk of disease progression and mortality in breast cancer patients following curative surgery. These findings underscore the potential importance of systematic psychological screening and integrated supportive care in the comprehensive management of breast cancer.

## Supplementary Information

Supplementary Material 1.

## Data Availability

Data will be made available on request.

## References

[1] Xiong X, Zheng LW, Ding Y, Chen YF, Cai YW, Wang LP, Huang L, Liu CC, Shao ZM, Yu KD. Breast cancer: pathogenesis and treatments. Signal Transduct Target Ther. 2025;10:49. 39966355 10.1038/s41392-024-02108-4 PMC11836418

[2] Bray F, Laversanne M, Sung H, Ferlay J, Siegel RL, Soerjomataram I, Jemal A. Global cancer statistics 2022: GLOBOCAN estimates of incidence and mortality worldwide for 36 cancers in 185 countries. CA Cancer J Clin. 2024;74:229–63. 38572751 10.3322/caac.21834

[3] Britt KL, Cuzick J, Phillips KA. Key steps for effective breast cancer prevention. Nat Rev Cancer. 2020;20:417–36. 32528185 10.1038/s41568-020-0266-x

[4] Plevritis SK, Munoz D, Kurian AW, Stout NK, Alagoz O, Near AM, Lee SJ, van den Broek JJ, Huang X, Schechter CB, et al. Association of screening and treatment with breast cancer mortality by molecular subtype in US Women, 2000–2012. JAMA. 2018;319:154–64. 29318276 10.1001/jama.2017.19130 PMC5833658

[5] Siegel RL, Kratzer TB, Giaquinto AN, Sung H, Jemal A. Cancer statistics, 2025. CA Cancer J Clin. 2025;75:10–45. 39817679 10.3322/caac.21871 PMC11745215

[6] Gulati M, Mulvagh SL. The connection between the breast and heart in a woman: breast cancer and cardiovascular disease. Clin Cardiol. 2018;41:253–7. 29446841 10.1002/clc.22886 PMC6489752

[7] Abdel-Qadir H, Austin PC, Lee DS, Amir E, Tu JV, Thavendiranathan P, Fung K, Anderson GM. A Population-Based study of cardiovascular mortality following Early-Stage breast cancer. JAMA Cardiol. 2017;2:88–93. 27732702 10.1001/jamacardio.2016.3841

[8] Phillips KA, Osborne RH, Giles GG, Dite GS, Apicella C, Hopper JL, Milne RL. Psychosocial factors and survival of young women with breast cancer: a population-based prospective cohort study. J Clin Oncol. 2008;26:4666–71. 18824713 10.1200/JCO.2007.14.8718 PMC2653129

[9] Groenvold M, Petersen MA, Idler E, Bjorner JB, Fayers PM, Mouridsen HT. Psychological distress and fatigue predicted recurrence and survival in primary breast cancer patients. Breast Cancer Res Treat. 2007;105:209–19. 17203386 10.1007/s10549-006-9447-x

[10] Riggio AI, Varley KE, Welm AL. The lingering mysteries of metastatic recurrence in breast cancer. Br J Cancer. 2021;124:13–26. 33239679 10.1038/s41416-020-01161-4 PMC7782773

[11] Stuart-Harris R, Dahlstrom JE, Gupta R, Zhang Y, Craft P, Shadbolt B. Recurrence in early breast cancer: analysis of data from 3,765 Australian women treated between 1997 and 2015. Breast. 2019;44:153–9. 30785024 10.1016/j.breast.2019.02.004

[12] Carter CL, Allen C, Henson DE. Relation of tumor size, lymph node status, and survival in 24,740 breast cancer cases. Cancer. 1989;63:181–7. 2910416 10.1002/1097-0142(19890101)63:1<181::aid-cncr2820630129>3.0.co;2-h

[13] Chen SJ, Chang CH, Chen KC, Liu CY. Association between depressive disorders and risk of breast cancer recurrence after curative surgery. Med (Baltim). 2016;95:e4547. 10.1097/MD.0000000000004547 PMC5370804 27537578

[14] Pinquart M, Duberstein PR. Depression and cancer mortality: a meta-analysis. Psychol Med. 2010;40:1797–810. 20085667 10.1017/S0033291709992285 PMC2935927

[15] Satin JR, Linden W, Phillips MJ. Depression as a predictor of disease progression and mortality in cancer patients: a meta-analysis. Cancer. 2009;115:5349–61. 19753617 10.1002/cncr.24561

[16] Soqia J, Al-Shafie M, Agha LY, Alameer MB, Alhomsi D, Saadoun R, Saifo M. Depression, anxiety and related factors among Syrian breast cancer patients: a cross-sectional study. BMC Psychiatry. 2022;22:796. 36528568 10.1186/s12888-022-04469-y PMC9759902

[17] Sharma A, Sriyuktasuth A, Phligbua W, Vongsirimas N. Psychological distress among breast cancer survivor and their spousal caregiver. J Nepal Health Res Counc. 2024;22:502–8. 39923162 10.33314/jnhrc.v22i03.4881

[18] Wang X, Wang N, Zhong L, Wang S, Zheng Y, Yang B, Zhang J, Lin Y, Wang Z. Prognostic value of depression and anxiety on breast cancer recurrence and mortality: a systematic review and meta-analysis of 282,203 patients. Mol Psychiatry. 2020;25:3186–97. 32820237 10.1038/s41380-020-00865-6 PMC7714689

[19] Eskelinen M, Korhonen R, Selander T, Ollonen P. Beck depression inventory as a predictor of Long-term outcome among patients admitted to the breast cancer diagnosis unit: A 25-year cohort study in Finland. Anticancer Res. 2017;37:819–24. 28179336 10.21873/anticanres.11383

[20] Juan Z, Chen J, Ding B, Yongping L, Liu K, Wang L, Le Y, Liao Q, Shi J, Huang J, et al. Probiotic supplement attenuates chemotherapy-related cognitive impairment in patients with breast cancer: a randomised, double-blind, and placebo-controlled trial. Eur J Cancer. 2022;161:10–22. 34896904 10.1016/j.ejca.2021.11.006

[21] Leentjens AF, Dujardin K, Marsh L, Martinez-Martin P, Richard IH, Starkstein SE, Weintraub D, Sampaio C, Poewe W, Rascol O, et al. Anxiety rating scales in parkinson’s disease: critique and recommendations. Mov Disord. 2008;23:2015–25. 18792121 10.1002/mds.22233

[22] Sun W, Shen J, Sun R, Zhou D, Li H. Establishment and validation of a predictive model for Post-Treatment anxiety based on patient attributes and Pre-Treatment anxiety scores. Psychol Res Behav Manag. 2023;16:3883–94. 37745270 10.2147/PRBM.S425055 PMC10517682

[23] Nelson CJ, Cho C, Berk AR, Holland J, Roth AJ. Are gold standard depression measures appropriate for use in geriatric cancer patients? A systematic evaluation of self-report depression instruments used with geriatric, cancer, and geriatric cancer samples. J Clin Oncol. 2010;28:348–56. 19996030 10.1200/JCO.2009.23.0201 PMC2815722

[24] Zhao YJ, Gui PP, Xu JJ, Guo T, Li J, Wang J, Wang G, Meng L. Exploring the differences in psychometric properties of commonly used self-rating depression scales across various populations in china: A quantitative systematic review. Asian J Psychiatr. 2025;111:104635. 40752070 10.1016/j.ajp.2025.104635

[25] Wang YH, Li JQ, Shi JF, Que JY, Liu JJ, Lappin JM, Leung J, Ravindran AV, Chen WQ, Qiao YL, et al. Depression and anxiety in relation to cancer incidence and mortality: a systematic review and meta-analysis of cohort studies. Mol Psychiatry. 2020;25:1487–99. 31745237 10.1038/s41380-019-0595-x

[26] Shim EJ, Lee JW, Cho J, Jung HK, Kim NH, Lee JE, Min J, Noh WC, Park SH, Kim YS. Association of depression and anxiety disorder with the risk of mortality in breast cancer: A National health insurance service study in Korea. Breast Cancer Res Treat. 2020;179:491–8. 31673880 10.1007/s10549-019-05479-3

[27] Iglay K, Santorelli ML, Hirshfield KM, Williams JM, Rhoads GG, Lin Y, Demissie K. Impact of preexisting mental illness on All-Cause and breast cancer-Specific mortality in elderly patients with breast cancer. J Clin Oncol. 2017;35:4012–8. 28934000 10.1200/JCO.2017.73.4947

[28] Bucsek MJ, Qiao G, MacDonald CR, Giridharan T, Evans L, Niedzwecki B, Liu H, Kokolus KM, Eng JW, Messmer MN, et al. β-Adrenergic signaling in mice housed at standard temperatures suppresses an effector phenotype in CD8(+) T cells and undermines checkpoint inhibitor therapy. Cancer Res. 2017;77:5639–51. 28819022 10.1158/0008-5472.CAN-17-0546 PMC5645237

[29] Yang H, Xia L, Chen J, Zhang S, Martin V, Li Q, Lin S, Chen J, Calmette J, Lu M, et al. Stress-glucocorticoid-TSC22D3 axis compromises therapy-induced antitumor immunity. Nat Med. 2019;25:1428–41. 31501614 10.1038/s41591-019-0566-4

[30] Zhang S, Yu F, Che A, Tan B, Huang C, Chen Y, Liu X, Huang Q, Zhang W, Ma C, et al. Neuroendocrine regulation of Stress-Induced T cell dysfunction during lung cancer immunosurveillance via the Kisspeptin/GPR54 signaling pathway. Adv Sci (Weinh). 2022;9:e2104132. 35224894 10.1002/advs.202104132 PMC9069377

