# Supplementary material for: The effects of emotional distress on the postoperative survival and progression in patients with breast cancer: a prospective observational study
Source: World J Surg Oncol. 2025 Dec 30;24:39. doi: 10.1186/s12957-025-04162-w (PMC12809896; doi:10.1186/s12957-025-04162-w)
Supplement: Supplementary file 1 — Supplementary Material 1. [file 12957_2025_4162_MOESM1_ESM.docx]

Supplementary Table S1: Proportional hazards assumption test results for univariable Cox regression models

|  | PFS | | | OS | | |
| --- | --- | --- | --- | --- | --- | --- |
|  | chisq | df | *p* | chisq | df | *p* |
| Depression | <0.01 | 1 | 0.999 | <0.01 | 1 | 0.996 |
| Anxiety | 2.01 | 1 | 0.155 | 0.78 | 1 | 0.378 |
| Emotional Distress | 0.12 | 1 | 0.733 | 0.03 | 1 | 0.873 |
| Note: In the proportional hazards assumption test, chisq = chi-square statistic, df = degrees of freedom. A p-value < 0.05 indicates that the variable does not satisfy the proportional hazards assumption. | | | | | | |

| Supplementary Table S2: Proportional hazards assumption test results for multivariable Cox regression models | | | | | | |
| --- | --- | --- | --- | --- | --- | --- |
|  | PFS | | | OS | | |
|  | chisq | df | *p* | chisq | df | *p* |
| Multivariable Cox regression model for depression | | | | | | |
| Depression | 0.15 | 1 | 0.698 | 0.28 | 1 | 0.595 |
| Treatment | 0.60 | 1 | 0.441 | 0.01 | 1 | 0.915 |
| Age | 0.97 | 1 | 0.325 | 0.49 | 1 | 0.486 |
| BMI | 0.11 | 1 | 0.746 | 0.89 | 1 | 0.345 |
| Molecular subtype | 5.51 | 2 | 0.064 | 5.27 | 2 | 0.072 |
| TNM Stage | 3.16 | 1 | 0.075 | 1.37 | 1 | 0.242 |
| GLOBAL | 13.81 | 7 | 0.055 | 7.95 | 7 | 0.337 |
| Multivariable Cox regression model for anxiety | | | | | | |
| Anxiety | 1.53 | 1 | 0.216 | 0.34 | 1 | 0.563 |
| Treatment | 0.08 | 1 | 0.779 | 0.05 | 1 | 0.824 |
| Age | 0.61 | 1 | 0.433 | 1.08 | 1 | 0.298 |
| BMI | 0.01 | 1 | 0.906 | 0.36 | 1 | 0.547 |
| Molecular subtype | 4.04 | 2 | 0.132 | 3.61 | 2 | 0.164 |
| TNM Stage | 3.39 | 1 | 0.066 | 1.76 | 1 | 0.185 |
| GLOBAL | 12.48 | 7 | 0.086 | 8.90 | 7 | 0.26 |
| Multivariable Cox regression model for emotional distress | | | | | | |
| Emotional Distress | <0.01 | 1 | 0.954 | 0.10 | 1 | 0.757 |
| Treatment | 0.33 | 1 | 0.566 | 0.00 | 1 | 0.987 |
| Age | 0.99 | 1 | 0.321 | 0.59 | 1 | 0.441 |
| BMI | 0.01 | 1 | 0.918 | 0.70 | 1 | 0.402 |
| Molecular subtype | 5.01 | 2 | 0.082 | 4.74 | 2 | 0.093 |
| TNM Stage | 3.41 | 1 | 0.065 | 1.49 | 1 | 0.222 |
| GLOBAL | 13.22 | 7 | 0.067 | 8.32 | 7 | 0.305 |

Note: The Schoenfeld residuals test was used to assess the proportional hazards assumption. A p-value < 0.05 indicates a significant violation of the assumption. Molecular subtype was treated as a categorical variable with 3 levels (df=2); other variables were treated as categorical (df=1) or continuous (df=1) as appropriate. GLOBAL represents the model-wide test of the proportional hazards assumption.

| Supplementary Table S3: Summary statistics of IPTW weights | | | | | | | | |
| --- | --- | --- | --- | --- | --- | --- | --- | --- |
| Variable | N | Mean | SD | Median | Min | Max | Q1 | Q3 |
| IPTW for Depression | 159 | 1.00 | 0.21 | 0.97 | 0.28 | 2.25 | 0.95 | 1.01 |
| IPTW for Anxiety | 159 | 1.01 | 0.26 | 0.98 | 0.33 | 3.05 | 0.94 | 1.03 |
| IPTW for ED | 159 | 1.00 | 0.26 | 0.96 | 0.37 | 2.60 | 0.91 | 1.05 |
| Note: SD, standard deviation; Q1, first quartile; Q3, third quartile. Extreme weights were defined as >10 or <0.1. | | | | | | | | |

| Supplementary Table S4: Covariate balance (SMD) before and after IPTW adjustment (Depression subgroup) | | | | | | | | |
| --- | --- | --- | --- | --- | --- | --- | --- | --- |
|  | Before IPTW | | | | After IPTW | | | |
|  | Non- Depression | Depression | *p* | SMD | Non- Depression | Depression | *p* | SMD |
| Age,mean (sd), y | 46.22±8.42 | 42.89±8.58 | 0.109 | 0.39 | 45.87±8.42 | 46.66±8.70 | 0.738 | 0.09 |
| BMI,mean (sd),kg/m² | 23.07±3.04 | 22.76±2.45 | 0.672 | 0.11 | 23.04±3.04 | 23.68±2.39 | 0.309 | 0.24 |
| Treatment, n (%) |  |  | 0.646 | 0.17 |  |  | 0.936 | 0.02 |
| Placebo | 71 (50.7) | 8 (42.1) |  |  | 69.4 (49.6) | 10.3 (50.8) |  |  |
| Probiotics | 69 (49.3) | 11 (57.9) |  |  | 70.3 (50.4) | 10.0 (49.2) |  |  |
| Molecular subtype, n (%) |  |  | 0.733^#^ | 0.20 |  |  | 0.970^#^ | 0.07 |
| Tri_Negative | 19 (13.6) | 2 (10.5) |  |  | 18.6 (13.3) | 2.6 (12.7) |  |  |
| HER-2-Postive | 46 (32.9) | 5 (26.3) |  |  | 44.8 (32.1) | 7.1 (35.3) |  |  |
| Luminal A | 75 (53.6) | 12 (63.2) |  |  | 76.3 (54.6) | 10.5 (52.0) |  |  |
| TNM Stage, n (%) |  |  | 0.141 | 0.41 |  |  | 0.651 | 0.11 |
| І | 39 (27.9) | 9 (47.4) |  |  | 41.8 (29.9) | 5.1 (25.0) |  |  |
| П&Ш | 101 (72.1) | 10 (52.6) |  |  | 97.9 (70.1) | 15.2 (75.0) |  |  |
| *Note: Continuous variables are presented as mean±SD (normal distribution) or median (IQR) (non-normal distribution); categorical variables as n (%). Comparisons were performed using independent samples t-test, Mann-Whitney U test, χ² test, or Fisher’s exact test (#) as appropriate. SMD, standardized mean difference; \|SMD\| < 0.25 indicates acceptable balance. p < 0.05. | | | | | | | | |

| Supplementary Table S5: Covariate balance (SMD) before and after IPTW adjustment (Anxiety subgroup) | | | | | | | | |
| --- | --- | --- | --- | --- | --- | --- | --- | --- |
|  | Before IPTW | | | | After IPTW | | | |
|  | Non- Anxiety | Anxiety | p | SMD | Non- Anxiety | Anxiety | p | SMD |
| Age, median (IQR), y | 46.00 (39.25,53.00) | 45.00 (38.00,47.00) | 0.184 | 0.39 | 46.00 (39.00,52.17) | 45.84 (38.00,48.55) | 0.652 | 0.15 |
| BMI, mean (sd),kg/m² | 23.05±3.01 | 22.86±2.54 | 0.824 | 0.07 | 23.03±3.00 | 22.63±2.43 | 0.604 | 0.15 |
| Treatment, n (%) |  |  | 0.579 | 0.25 |  |  | 0.719 | 0.13 |
| Placebo | 74 (50.7) | 5 (38.5) |  |  | 72.7 (49.8) | 6.9 (56.0) |  |  |
| Probiotics | 72 (49.3) | 8 (61.5) |  |  | 73.4 (50.2) | 5.5 (44.0) |  |  |
| Molecular subtype, n (%) |  |  | 0.768# | 0.22 |  |  | 0.418# | 0.44 |
| Tri Negative | 19 (13.0) | 2 (15.4) |  |  | 19.4 (13.3) | 2.0 (16.3) |  |  |
| HER-2-Postive | 48 (32.9) | 3 (23.1) |  |  | 46.7 (32.0) | 1.7 (13.8) |  |  |
| Luminal A | 79 (54.1) | 8 (61.5) |  |  | 80.0 (54.8) | 8.7 (69.9) |  |  |
| TNM Stage, n (%) |  |  | 0.024*# | 0.73 |  |  | 0.986# | 0.01 |
| І | 40 (27.4) | 8 (61.5) |  |  | 44.1 (30.2) | 3.8 (30.4) |  |  |
| П&Ш | 106 (72.6) | 5 (38.5) |  |  | 101.9 (69.8) | 8.6 (69.6) |  |  |
| *Note: Continuous variables are presented as mean±SD (normal distribution) or median (IQR) (non-normal distribution); categorical variables as n (%). Comparisons were performed using independent samples t-test, Mann-Whitney U test, χ² test, or Fisher’s exact test (#) as appropriate. SMD, standardized mean difference; \|SMD\| < 0.25 indicates acceptable balance. p < 0.05. | | | | | | | | |

| Supplementary Table S6: Covariate balance (SMD) before and after IPTW adjustment (Emotional Distress subgroup) | | | | | | | | |
| --- | --- | --- | --- | --- | --- | --- | --- | --- |
|  | Before IPTW | | | | After IPTW | | | |
|  | NO ED | ED | *p* | SMD | NO ED | ED | *p* | SMD |
| Age, median (IQR), y | 46.00 (39.75,53.00) | 45.00 (38.00,49.00) | 0.228 | 0.26 | 46.00 (39.00, 52.00) | 47.00 (39.67, 52.50) | 0.836 | 0.04 |
| BMI, mean (sd),kg/m² | 23.05±3.05 | 22.92±2.61 | 0.836 | 0.05 | 23.04±3.05 | 23.34 ±2.56 | 0.611 | 0.11 |
| Treatment, n (%) |  |  | 0.699 | 0.13 |  |  | 0.911 | 0.03 |
| Placebo | 67 (50.8) | 12 (44.4) |  |  | 65.4 (49.6) | 14.0 (51.0) |  |  |
| Probiotics | 65 (49.2) | 15 (55.6) |  |  | 66.4 (50.4) | 13.5 (49.0) |  |  |
| Molecular subtype, n (%) |  |  | 0.639# | 0.20 |  |  | 0.967# | 0.06 |
| Tri_Negative | 18 (13.6) | 3 (11.1) |  |  | 17.6 (13.3) | 3.4 (12.3) |  |  |
| HER-2-Postive | 44 (33.3) | 7 (25.9) |  |  | 41.9 (31.8) | 8.2 (29.8) |  |  |
| Luminal A | 70 (53.0) | 17 (63.0) |  |  | 72.2 (54.8) | 15.9 (57.9) |  |  |
| TNM Stage, n (%) |  |  | 0.014* | 0.56 |  |  | 0.854 | 0.04 |
| І | 34 (25.8) | 14 (51.9) |  |  | 39.5 (30.0) | 7.8 (28.2) |  |  |
| П&Ш | 98 (74.2) | 13 (48.1) |  |  | 92.3 (70.0) | 19.7 (71.8) |  |  |
| *Note: Continuous variables are presented as mean±SD (normal distribution) or median (IQR) (non-normal distribution); categorical variables as n (%). Comparisons were performed using independent samples t-test, Mann-Whitney U test, χ² test, or Fisher’s exact test (#) as appropriate. SMD, standardized mean difference; \|SMD\| < 0.25 indicates acceptable balance. p < 0.05. | | | | | | | | |

| Supplementary Table S7: Variance Inflation Factors (VIF) for covariates in IPTW models | | | |
| --- | --- | --- | --- |
|  | VIF for Depression | VIF for Anxiety | VIF for ED |
| Treatment | 1.01 | 1.02 | 1.01 |
| Age | 1.07 | 1.08 | 1.07 |
| BMI | 1.03 | 1.04 | 1.04 |
| Molecular subtype | 1.02 | 1.02 | 1.02 |
| TNM Stage | 1.02 | 1.02 | 1.02 |
| Note: VIF, Variance Inflation Factor; VIF > 5 indicates significant collinearity. All covariates had VIF < 1.1, confirming no collinearity. | | | |

| Supplementary Table S8: Multicollinearity assessment (VIF) in multivariable Cox regression models | | |
| --- | --- | --- |
|  | PFS | OS |
| Multivariable Cox regression model for depression |  |  |
| Depression | 1.38 | 2.13 |
| Treatment | 1.69 | 2.21 |
| Age | 1.80 | 2.07 |
| BMI | 1.41 | 1.25 |
| Molecular subtype | 2.11 | 1.58 |
| TNM Stage | 1.83 | 1.54 |
| Multivariable Cox regression model for anxiety |  |  |
| Anxiety | 1.48 | 1.27 |
| Treatment | 1.42 | 1.63 |
| Age | 1.68 | 1.21 |
| BMI | 1.26 | 1.23 |
| Molecular subtype | 2.32 | 1.28 |
| TNM Stage | 1.79 | 1.53 |
| Multivariable Cox regression model for ED |  |  |
| ED | 1.68 | 2.76 |
| Treatment | 1.89 | 2.62 |
| Age | 1.52 | 2.29 |
| BMI | 1.34 | 1.29 |
| Molecular subtype | 2.31 | 1.75 |
| TNM Stage | 2.05 | 1.85 |
| Note: In the proportional hazards assumption test, chisq = chi-square statistic, df = degrees of freedom. A p-value < 0.05 indicates that the variable does not satisfy the proportional hazards assumption. | | |

| Table 9 Results of Sensitivity Analysis | | | | |
| --- | --- | --- | --- | --- |
|  | PFS | | OS | |
|  | HR (95%CI) | *P* | HR (95%CI) | *P* |
| Depression | | | | |
| No | Reference |  | Reference |  |
| Yes | 5.253 (1.54,17.87) | 0.008 | 8.420 (2.45,28.94) | 0.001 |
| Anxiety | | | | |
| No | Reference |  | Reference |  |
| Yes | 2.372 (0.46,12.34) | 0.305 | 2.506 (0.78,8.07) | 0.124 |
| ED | | | | |
| No | Reference |  | Reference |  |
| Yes | 3.691 (1.04,13.15) | 0.044 | 6.466 (1.78,23.51) | 0.005 |
| Note: Models were adjusted for treatment assignment, age, BMI, tt(TNM Stage-II/III), tt(Molecular-HER2), and tt(Molecular-Luminal), where tt = covariate × log(time + 20). All analyses were weighted by the inverse probability of depression/anxiety/emotional distress status. | | | | |

| Table S10: IPTW-weighted progression-free survival (PFS) and overall survival (OS) rates at 1, 3, 5, and 7 years by psychological status | | | | |
| --- | --- | --- | --- | --- |
|  | 1-years | 3-years | 5-years | 7-years |
| **PFS survival rate (%)** | | | | |
| **Depression** |  |  |  |  |
| No | 140 (100%) | 132 (94.6%) | 130 (93.3%) | 125 (89.7%) |
| Yes | 20 (100%) | 16 (78.3%) | 14 (66.7%) | 14 (66.7%) |
| **Anxiety** |  |  |  |  |
| No | 146 (100%) | 138 (94.1%) | 135 (92.2%) | 130 (88.8%) |
| Yes | 12 (100%) | 11 (88.1%) | 11 (85.5%) | 11 (85.5%) |
| Emotional Distress |  |  |  |  |
| No | 132 (100%) | 124 (94.5%) | 123 (93.1%) | 118 (89.3%) |
| Yes | 27 (100%) | 23 (84.6%) | 21 (76.9%) | 21 (76.9%) |
| **OS survival rate (%)** | | | | |
| **Depression** |  |  |  |  |
| No | 140 (100%) | 134 (96%) | 134 (96%) | 131 (93.9%) |
| Yes | 20 (100%) | 16 (78.3%) | 16 (78.3%) | 14 (68.8%) |
| **Anxiety** |  |  |  |  |
| No | 146 (100%) | 139 (95.4%) | 139 (95.4%) | 136 (92.8%) |
| Yes | 12 (100%) | 11 (88.1%) | 11 (88.1%) | 11 (88.1%) |
| Emotional Distress |  |  |  |  |
| No | 132 (100%) | 126 (95.9%) | 126 (95.9%) | 124 (93.7%) |
| Yes | 27 (100%) | 23 (84.6%) | 23 (84.6%) | 22 (78.5%) |
|  | | | | |
